# Immune checkpoint inhibitors associated cardiovascular immune-related adverse events

**DOI:** 10.3389/fimmu.2024.1340373

**Published:** 2024-02-05

**Authors:** Wonyoung Jo, Taejoon Won, Abdel Daoud, Daniela Čiháková

**Affiliations:** ^1^ Department of Biomedical Engineering, Johns Hopkins University, Whiting School of Engineering, Baltimore, MD, United States; ^2^ Department of Pathobiology, University of Illinois Urbana-Champaign, College of Veterinary Medicine, Urbana, IL, United States; ^3^ W. Harry Feinstone Department of Molecular Microbiology and Immunology, Johns Hopkins University, Bloomberg School of Public Health, Baltimore, MD, United States; ^4^ Department of Pathology, Johns Hopkins University, School of Medicine, Baltimore, MD, United States

**Keywords:** immune checkpoint inhibitors, myocarditis, atherosclerosis, CTLA-4, PD-1, LAG-3, TIM-3, TIGIT

## Abstract

Immune checkpoint inhibitors (ICIs) are specialized monoclonal antibodies (mAbs) that target immune checkpoints and their ligands, counteracting cancer cell-induced T-cell suppression. Approved ICIs like cytotoxic T-lymphocyte antigen-4 (CTLA-4), programmed death-1 (PD-1), its ligand PD-L1, and lymphocyte activation gene-3 (LAG-3) have improved cancer patient outcomes by enhancing anti-tumor responses. However, some patients are unresponsive, and others experience immune-related adverse events (irAEs), affecting organs like the lung, liver, intestine, skin and now the cardiovascular system. These cardiac irAEs include conditions like myocarditis, atherosclerosis, pericarditis, arrhythmias, and cardiomyopathy. Ongoing clinical trials investigate promising alternative co-inhibitory receptor targets, including T cell immunoglobulin and mucin domain-containing protein 3 (Tim-3) and T cell immunoreceptor with immunoglobulin and ITIM domain (TIGIT). This review delves into the mechanisms of approved ICIs (CTLA-4, PD-1, PD-L1, and LAG-3) and upcoming options like Tim-3 and TIGIT. It explores the use of ICIs in cancer treatment, supported by both preclinical and clinical data. Additionally, it examines the mechanisms behind cardiac toxic irAEs, focusing on ICI-associated myocarditis and atherosclerosis. These insights are vital as ICIs continue to revolutionize cancer therapy, offering hope to patients, while also necessitating careful monitoring and management of potential side effects, including emerging cardiac complications.

## 1 Introduction

Remarkable advances have been made in recent years in cancer treatment (1). Immunotherapy represents a category of cancer treatment that harnesses the immune system’s elements to combat tumor cells (2). Techniques like adoptive cell transfer (ACT) and immune checkpoint inhibitors (ICIs) fall under this approach, offering promising methods in the fight against cancer (3). This innovative approach, either administered alone or in combination with conventional treatments like radiotherapy and chemotherapy, has become a prevailing standard for numerous cancers, exhibiting considerable success (4). A particularly promising strategy to trigger therapeutic anti-tumor immunity involves obstructing immune checkpoints (5).

The balance between co-stimulatory and inhibitory signals governs the ultimate amplitude and quality of the T-cell response, triggered by antigen recognition via the T-cell receptor (TCR) (5). The immune system relies on inhibitory pathways, known as immune checkpoints, to maintain self-tolerance and regulate the duration and strength of the immune response in peripheral tissues, thus minimizing collateral tissue damage (5). Unfortunately, tumor cells exploit these inhibitory molecules by expressing immune checkpoint proteins as a means to induce immune resistance and T-cell exhaustion (3). Given that ligand-receptor interactions initiate many immune checkpoints, these can be readily blocked by antibodies or modulated through recombinant forms of ligands and receptors (5). The treatment involving ICIs, particularly targeting cytotoxic T lymphocyte-associated antigen-4 (CTLA-4), programmed death-1 (PD-1), programmed death ligand-1 (PD-L1), and lymphocyte activation gene-3 (LAG-3), has been proven effective in activating anti-tumor T-cell activity and dynamically regulating the anti-tumor immune response (6).

However, despite the promising outcomes observed with immunotherapy in certain cancers, not all patients exhibit a favorable response to ICIs, and the overall response rate (ORR) is influenced by tumor type and specific drugs, ranging from 10.9% for single-agent ipilimumab (monoclonal antibody (mAbs) targeting CTLA-4, anti-CTLA-4 mAb) in previously treated melanoma to 69% for pembrolizumab (anti-PD-1 mAb) in relapsed/refractory classic Hodgkin’s lymphoma (7–9). Additionally, some patients may develop resistance to these treatments over time (10). Furthermore, a new set of side effects termed irAEs, immune-related adverse events, have been associated with ICI therapy (11). These irAEs manifest as autoimmune conditions affecting various organs throughout the body following ICI administration, and they have distinct characteristics compared to non-ICI therapy-related autoimmune diseases (11). Among the reported cases of irAEs in cancer patients treated with ICI, cardiotoxicity has emerged as a concerning issue in recent years (12). Although immune-related cardiotoxicity is less frequent than other irAEs, it is often fatal (13). Studies analyzing safety databases have identified potential ICI-associated cardiac toxicities, including myocarditis, cardiomyopathy, conduction defects (heart block), vasculitis, atrial and ventricular arrhythmias, pericarditis/pericardial effusion, venous thrombosis, acute coronary syndrome, tachycardia, hypotension, and cardiac dysfunction (13–23). Significant focus of ICI cardiotoxicity research is centered on myocarditis, a rare cardiac irAE with notable mortality (24, 25). Recently attention has also expanded towards ICI-associated atherosclerosis, given the ICI role in accelerating atherosclerotic cardiovascular disease with substantial implications for long-term vascular toxicity (26). In this review, ICI-myocarditis and atherosclerosis are chosen as the primary focus, emphasizing the clinical significance of these two major cardiotoxic irAEs. This strategic selection underscores the critical need for a comprehensive exploration and management of cardiovascular irAEs associated with ICI therapy.

In an effort to increase the percentage of responsive cancers to these therapies, researchers are exploring novel pathways and molecules to enhance the response and effectiveness of ICI therapy (27). Among the promising options are ICIs targeting T-cell immunoglobulin and mucin-domain-containing-3 (Tim-3) and T-cell immunoglobulin and ITIM domain (TIGIT), which show potential for treating solid tumors and are currently under active investigation in clinical trials (6). This review aims to provide a comprehensive overview of the mechanisms and cardiotoxicity associated with Food and Drug Administration (FDA)-approved ICIs. Additionally, we focus on next-generation ICIs, Tim-3 and TIGIT inhibitors, exploring their influence on T-cell function, role in cancer treatment, and potential for cardiac irAEs.

## 2 FDA-approved immunotherapy targets: CTLA-4, PD-1, and LAG-3

The mAbs that inhibit immune checkpoints have displayed remarkable efficacy in clinical trials and have made significant advancements in oncology (7, 28–32). While targeted therapies often result in short-lived clinical responses due to the development of drug resistance within a few months, immune checkpoint blockade therapies have demonstrated durable clinical responses (7, 32, 33). Some patients have experienced prolonged periods without cancer progression, spanning several years (7, 32, 33). These ICIs, including ipilimumab (anti-CTLA-4 mAb), pembrolizumab, nivolumab, cemiplimab (anti-PD-1 mAb), as well as atezolizumab, avelumab, and durvalumab (anti-PD-L1 mAb), have become standard in clinical practice (7, 33–37). They have demonstrated remarkable effectiveness against diverse types of cancer that was previously unparalleled (7, 33–37). In recent developments, two agents directed at LAG-3 (eftilagimod alpha and relatlimab, anti-LAG-3) have received approval for use in combination with anti-PD-1 to treat advanced solid tumors due to their crucial role in modulating the immune system (38).

### 2.1 Mechanism and signaling pathways orchestrated by CTLA-4

CTLA-4, also referred to as CD152, represents one of the initial negative modulators that have been targeted in clinical settings (39). It is predominantly present in conventional T cells following activation and Foxp3+ regulatory T lymphocytes (Treg) cells, and its main function is to regulate the intensity of early T cell activation (40–43). The use of ipilimumab (anti-CTLA-4 mAb) either alone or in combination with nivolumab (anti-PD-1 mAb) is currently approved for the treatment of melanoma, colorectal cancer (CRC), hepatocellular carcinoma (HCC), non-small cell lung cancer (NSCLC), and renal cell carcinoma (RCC) (44).

CTLA-4 functions by counteracting CD28, a co-stimulatory receptor on T cells (45–47), and shares ligands [CD80 (B7.1) and CD86 (B7.2)] with CD28 (48–52). While the exact mechanisms of CTLA-4 action are debated (5), one theory proposes that its higher affinity for CD80 and CD86 dampens T cell activation by outcompeting CD28 for binding (53–58). Upon binding to CD80 and CD86 on antigen-presenting cells, CTLA-4 triggers inhibitory reactions via protein phosphatases SHP2 and PP2A, resulting in immune suppression (59), including the blockade of T lymphocyte response, reduced T lymphocyte proliferation, increased Treg activity, decreased cytokine secretion, and overall immunosuppression (60–62). Furthermore, it has been shown that ipilimumab (anti-CTLA-4 mAb) selectively depletes CTLA-4+ FOXP3+ Treg cells via antibody-dependent cell-mediated (ADCC) cytotoxicity (40).

### 2.2 Mechanism and signaling pathways orchestrated by PD-1/PD-L1

PD-1 and PD-L1 inhibitors function by blocking inhibitory signals, thereby supporting the eradication of tumor cells through sustained activation of T lymphocytes (63, 64). PD-1 is expressed on various immune cells, including monocytes, T cells, B cells, dendritic cells (DCs), and tumor-infiltrating lymphocytes (TILs) (65, 66). On the other hand, PD-L1 is typically found on APCs and tumor cells (66). In human cancers, PD-1 has been predominantly detected in a wide range of malignancies, such as melanoma, lung cancer, RCC, head and neck cancer, bladder cancer, ovarian cancer, and gastrointestinal cancer (67).

In contrast to CTLA-4, which primarily regulates T-cell proliferation in lymph nodes during early immune responses, PD-1 plays a crucial role in limiting T cell activity in peripheral tissues during inflammatory responses and contributes to autoimmune control (68–74). PD-1 plays a significant role as an immune resistance mechanism within the tumor microenvironment (TME) (75–77). PD-1 expression counteracts positive signaling events initiated by TCR and CD28 interactions, leading to the inhibition of transcription factors vital for T cell activation, proliferation, effector functions, and survival (39). This includes suppression of activator protein 1 (AP-1), nuclear factor of activated T cells (NFAT), nuclear factor-κB (NF-κB), and anti-apoptotic proteins like Bcl-2 and Bcl-xL, impairing T-cell survival (78). Ligand activation of PD-1 triggers a negative feedback pathway, reducing cytokine production (59). Similarly, to CTLA-4, PD-1 is highly expressed on Treg cells, potentially enhancing their proliferation in the presence of its ligand (79).

### 2.3 Mechanism and signaling pathways orchestrated by LAG-3

Identified more than two decades ago as a CD4 homologue (80), LAG-3’s role as an immune checkpoint was clarified in 2005 when it was discovered to enhance Treg cell function (81, 82). Beyond its influence on Treg cells, LAG-3 independently inhibits CD8+ effector T cells (83). Its sole known ligand is MHC class II molecules, upregulated on certain epithelial cancers in response to interferon-γ (IFN-γ), also found on tumor-infiltrating macrophages and DCs (5). While the precise function of the LAG-3-MHC class II interaction in inhibiting T cell responses is not fully understood, LAG-3 antibodies that do not block this interaction have been observed to enhance T cell proliferation and improve effector cell functions *in vitro* and *in vivo* (5).

The specific molecular mechanisms underlying LAG-3 signaling and its interaction with other immune checkpoints are largely uncertain (84). However, LAG-3 has shown remarkable synergistic effects with PD-1, inhibiting immune responses in various scenarios (85, 86). Particularly, the combination therapy of relatlimab (anti-LAG-3 mAb; BMS-986016) with nivolumab (anti-PD-1 mAb) has demonstrated impressive clinical effectiveness in melanoma patients unresponsive to anti-PD-1/PD-L1 therapy (87). Moreover, LAG-3 expression was significantly higher in various cancers, including kidney renal clear cell carcinoma (KIRC), pancreatic adenocarcinoma (PAAD), skin cutaneous melanoma (SKCM), testicular germ cell tumors (TGCT), lymphoid neoplasm diffuse large B‐cell lymphoma (DLBC), and head and neck squamous cell carcinoma (HNSC), compared to their corresponding normal tissues (88). This suggests that targeting LAG-3 could have a substantial antitumor impact in these cancer types (88).

### 2.4 Application of CTLA-4/PD-1/LAG-3 blockade in clinical settings

Immunotherapy with ICIs offers numerous benefits compared to traditional cancer treatments (27). Clinical studies focusing on melanoma, RCC, squamous cell carcinoma, and NSCLC have shown remarkable increases in patient survival with both CTLA-4 and PD-1 checkpoint inhibitors compared to conventional chemotherapy (89). Particularly, ICIs have demonstrated the potential for durable responses and even the possibility of cure in metastatic diseases (27). Another advantage of ICI treatment is the availability of biomarkers to predict the response to therapy (27). These biomarkers help identify patients who are likely to benefit the most from immunotherapy, making it a more personalized and targeted approach (90).

As the inhibitory roles of CTLA-4 and PD-1 in immune responses, including antitumor responses, are distinct, their effectiveness in cancer treatment depends significantly on the cancer type and tumor size (27, 44). Notably, in melanoma, while CTLA-4 blockade with ipilimumab was the first treatment to extend overall survival in patients with advanced melanoma in a randomized setting, anti-PD-1 mAb treatment (nivolumab) demonstrated higher efficacy in patients with smaller tumors (7, 91–94). A phase 3 clinical trial directly comparing the two ICIs found that patients treated with nivolumab (anti-PD-1 mAb) had a better response rate (44%) and longer progression-free survival (6.9 months) than those treated with ipilimumab (anti-CTLA-4 mAb) (response rate of 19% and progression-free survival of 2.8 months) (95).

The combination of CTLA-4 and PD-1 blockers has been proposed to have a synergistic effect on activating anti-tumor immune responses, leading to increased response rates in patients (96). Numerous clinical studies have been conducted to assess the safety and efficacy of this combination in various cancer subtypes (97). Recent data indicates that the dual inhibition of PD-1 and CTLA-4 may enhance the activity observed with single-agent therapy by promoting the recruitment of peripheral T-cells and reducing resident Tregs (96). Clinical results have shown that when PD-1/PD-L1 inhibitors and CTLA-4 inhibitors are administered individually, the response rates are typically around 20-25% at best (95). However, when these inhibitors are combined, the response rate can reach up to 60%, with an associated survival benefit of 11.5 months (95). This significant increase in response rates and median survival times has been observed in melanoma and RCC, leading to the approval of the ipilimumab (anti-CTLA-4 mAb) and nivolumab (anti-PD-1 mAb) combination for the treatment of these cancers (98, 99).

Apart from CTLA-4 and PD-1/PD-L1, LAG-3 has emerged as a promising target for tumor immunotherapy (100). Currently, more than 80 clinical trials are ongoing worldwide to assess drug candidates targeting LAG-3 (100). However, the efficacy of relatlimab (anti-LAG-3 mAb) alone is limited, and it is typically utilized in combination with other checkpoint inhibitors like ipilimumab (anti-CTLA-4 mAb) or nivolumab (anti-PD-1 mAb) to achieve synergistic enhancement of efficacy (101). Especially noteworthy is the combination of relatlimab (anti-LAG-3) and nivolumab (anti-PD-1), which has shown promising preliminary efficacy in melanoma patients previously unresponsive to anti-PD-1/PD-L1 therapy (NCT01968109) (86, 87). As a result of these findings, the FDA approved relatlimab (anti-LAG-3 mAb) in combination with nivolumab (anti-PD-1 mAb) in March 2022, making it the first mAb to be approved for the treatment of unresectable or metastatic melanoma (100).

## 3 Immune-related adverse events arising from blockade of CTLA-4, PD-1, or LAG-3

In clinical settings, ICIs, despite their proven efficacy, are associated with numerous irAEs affecting various organ systems (102). Approximately 60% to 80% of individuals undergoing ICI treatment experience irAEs, with 13% to 23% encountering severe grade 3 to 4 adverse events as per Common Terminology Criteria for Adverse Events (102). Common mild side effects include symptoms like diarrhea, fatigue, itching, rash, nausea, and reduced appetite (89). Serious adverse responses encompass severe diarrhea, colitis, elevated alanine aminotransferase levels, pneumonitis-related inflammation, and interstitial nephritis (95, 103, 104). Patients may also experience exacerbation of existing autoimmune conditions or the emergence of new ones, such as type 1 diabetes mellitus (105–108). Severe adverse effects may necessitate treatment discontinuation, although subsequent positive responses could still occur (109). Particularly, specific treatment-related autoimmune reactions, like rashes and vitiligo, have shown a connection with improved disease prognosis, suggesting a potential convergence between autoimmune and anti-tumor immune responses (110).

Under normal physiological circumstances, PD-1 and CTLA-4 prevent autoimmunity and curb immune activation to safeguard against unwanted inflammation (89). Using therapeutic antibodies to inhibit these receptors for cancer treatment is linked to adverse effects similar to autoimmune responses (89). Clinical studies contrasting various ICIs observed a higher incidence of side effects in individuals undergoing anti-CTLA-4 treatment (27.3%) compared to those receiving anti-PD-1 treatment (16.7%) (95). Animal studies corroborate this, with CTLA-4-deficient mice experiencing spontaneous severe myocarditis and pancreatitis, while PD-1-deficient mice develop lupus-like proliferative arthritis and glomerulonephritis on C57BL/6 background and myocarditis on BALB/c background (71, 72, 111). Thus, the nature of inflammation and adverse reactions induced in these knockout mice depends on their specific genetic background (72, 111–113).

Combined CTLA-4 and PD-1 blockade has increased anti-cancer efficacy, but it may also result in heightened toxicity (44). In melanoma or relapsed small cell lung cancer patients, combined blockade led to more severe grade 3-4 adverse events (54% to 55%) compared to solely anti-CTLA-4 (24% to 27%) or anti-PD-1 (15% to 16%) treatment (114–116). Similarly, despite the therapeutic enhancement achieved through combined PD-1 and LAG-3 targeting, there is a potential for increased toxicity. A study in melanoma patients showed that relatlimab (anti-LAG-3 mAb) plus nivolumab (anti-PD-1 mAb) led to a higher occurrence of adverse events (81.1% vs. 69.9%) and grade 3–4 irAEs (18.9% vs. 9.7%) compared to nivolumab (anti-PD-1 mAb) alone, with a notable rise in hepatitis cases (3.9% vs. 1.1%) (117). Overall, 14.6% of patients receiving the combinatory treatment discontinued it due to irAEs, compared to 6.7% in the nivolumab (anti-PD-1 mAb)-only group (117).

ICIs, by intensifying T cell activation, can enhance anti-tumor immune reactions but may also perturb peripheral T cell tolerance, potentially resulting in hyperinflammatory responses and autoimmune conditions (118). irAEs are associated with the infiltration of various inflammatory immune cells into affected organs, often showing a significant increase in CD8+ lymphocytes and a markedly elevated CD8+/CD4+ ratio (119, 120).

### 3.1 Cardiovascular immune-related adverse events

The majority of irAEs are manageable during their early stages; however, approximately 10–17% of these instances result in fatal outcomes (24, 25). While the incidence of cardiovascular adverse events caused by ICIs is relatively low, the associated mortality rate is alarmingly high (111). The spotlight on cardiac irAEs began in 2016, with Johnson et al. documenting two instances of fatal myocarditis occurring after ICI treatment (121, 122). Subsequently, the spectrum of cardiovascular irAEs has expanded to include conditions such as myocardial infarction, atrioventricular (AV) block, various forms of arrhythmias (including supraventricular and ventricular), sudden cardiac death, Tako-Tsubo cardiomyopathy, non-inflammatory cardiomyopathy, pericarditis, pericardial effusion, ischemic stroke, venous thromboembolism and accelerated atherosclerosis (123).

#### 3.1.1 Heart failure

In a retrospective analysis encompassing 424 patients who underwent treatment involving at least one ICI, it was found that 62 individuals (14.6%) received diagnoses of new cardiovascular disorders subsequent to the commencement of ICI therapy (124). Within this group, 5.6% experienced the development of heart failure during ICI monotherapy (124). This rate escalated to 6.1% when a sequential administration of two ICIs was implemented (124). A similar pattern emerged from a recent meta-analysis, involving a patient cohort of 13,646 individuals who were administered anti-CTLA-4, anti-PD-1, and/or anti-PD-L1 therapies (125). In the subset of patients receiving ICI as a sole therapeutic approach, the incidence of cardiovascular adverse events stood at 3.1% (125). Remarkably, this incidence nearly doubled to 5.8% among patients who received dual immunotherapy (125). The introduction of chemotherapy alongside ICI did not substantially alter the incidence rate, which remained at 3.7% (125). This data aligns with findings from clinical trials, suggesting that CTLA-4 inhibition is prone to causing immune-related cardiotoxicity, inclusive of conditions like pericarditis and myocarditis (126, 127). Likewise, instances of myocarditis have been recorded following the administration of anti-PD-1 drugs such as nivolumab or pembrolizumab (128, 129). An independent meta-analysis conducted by Dolladille et al., involving 32,518 patients, reinforced the association between ICI usage and an elevated risk of myocarditis, pericardial diseases, heart failure, dyslipidemia, myocardial infarction, and cerebral arterial ischemia (130). Significantly, ICI-treated patients, heart failure was more frequently observed adverse events. The calculated “number needed to harm”, indicating the number of individuals who must be exposed to a specific risk factor or treatment for one person to experience a particular adverse event, was found to be 462 for myocarditis and only 260 for heart failure (130).

#### 3.1.2 Immune checkpoint inhibitor associated myocarditis

Myocarditis, characterized by inflammation of the heart muscle, has seen a remarkable increase in its association with ICIs in recent years (15, 131, 132). Several preclinical studies have demonstrated a correlation between ICI treatment and myocarditis, as illustrated in **Table 1** (133–138). Notably, the initial trials of ICIs did not include proactive screenings for myocarditis, which raises concerns that some cases may have gone undetected (139). It was reported that approximately 0.04% to 1.14% of cases involving ICI treatments are linked to myocarditis (18, 121). Myocarditis stands out as the most frequently reported cardiac irAE, primarily due to its significant morbidity rates (13, 16, 140). In contrast to other irAEs, it carries a significantly elevated mortality rate ranging from 25% to 50% (15, 16, 18, 122).

**Table 1:** Preclinical models for immune checkpoint inhibitor associated myocarditis studies.

Immune Checkpoint Inhibition	Myocarditis induction	Susceptible Mice Strain	Advantages	Limitations	Reference
Anti-PD-1 mAb & Anti-PD-L1 mAb	CVB3 infection	C3H/He (4-9 weeks)	- Use of clinically relevant virus	- High mortality - High biosafety standard required - Not relevant induction to clinical ICI-myocarditis	Nagai et al. Cardiovasc Res. 2007 (133)
Anti-PD-1 mAb & Anti-PD-L1 mAb PD-1^-/-^ mice	*T.cruzi* infection	C57BL/6 (6-8weeks)	- Use of clinically relevant pathogen	- Pathogen strain-dependent variability - Long-term model	Silva et al. Infect Immun. 2011 (134)
PD-L1/2^-/-^ mice	Crossed to cMy-mOVA mice and OT-I T cell transfer	C57BL/6 (8-12 weeks)	- Biosafe - Suitable to study T cell mediated cytotoxicity against cardiomyocytes	- Reactivity against non-cardiac antigen - *In vitro* T cell activation - Genetic not mAb induced model	Lichtman et al. Circulation. 2007 (135)
Pdcd-1^-/-^ mice	Crossed to MRL mice (prone to autoimmune disease)	C57BL/6	- Biosafe - Suitable to study side effects of anti-PD-1/PD-L1 therapy	- Multiorgan involvement - High mortality - Genetic not mAb induced model	Okazaki et al. Int Immunol. 2010 (136)
Pdcd-1^-/-^ mice	Crossed to CTLA4^+/-^ mice	C57BL/6	- Biosafe - Suitable to study side effects of anti-CTLA-4 therapy	- High mortality - Genetic not mAb induced model	Moslehi and Allison et al. Cancer Discov. 2021 (137)
Anti-PD-1 mAb	Anti-PD-1 mAb alone	A/J (8-12 weeks)	- Biosafe - Clinically relevant model to study ICI-myocarditis	- Relatively low incidence - Reliance on A/J mice	Cihakova et al. Cell Rep. 2022 (138)

cMy, cardiac myosin; CVB3, Coxsackievirus B3; mOVA, membrane-anchored ovalbumin; PD-1, programmed death-1; PD-L1, programmed death ligand-1; Pdcd1, programmed cell death protein 1; *T.cruzi, Trypanosoma cruzi*.

A risk factor for myocarditis associated with ICIs is the utilization of combined ICI therapy (121). For instance, the combined usage of nivolumab (anti-PD-1 mAb) and ipilimumab (anti-CTLA-4 mAb) leads to a 4.74-fold increase in the risk of myocarditis when contrasted with the utilization of nivolumab (anti-PD-1 mAb) as a standalone therapy (122). Similarly, recent trials involving combined nivolumab (anti-PD-1 mAb) and relatlimab (anti-LAG-3 mAb) therapy revealed slightly elevated instances of myocarditis in comparison to the usage of single nivolumab (anti-PD-1 mAb) therapy (1.7% versus 0.6%, respectively) (117). Furthermore, instances of myocarditis stemming from combination therapies tend to have higher severity and higher rate of fatality (15). One study revealed that the mortality rate associated with the combination of anti-PD-1/PD-L1 and anti-CTLA-4 therapies was 67%, in contrast to the 36% fatality rate observed in anti-PD-1/PD-L1 monotherapy (15). However, it is important to note that due to the study’s limited sample size of only 59 patients, the reported fatality rate may not accurately reflect the true extent of the risk.

##### 3.1.2.1 Mechanism of immune checkpoint inhibitor associated myocarditis: cardiac specific autoreactive T cells

Patients who develop myocarditis may present with a diverse range of symptoms, including chest pain, elevated cardiac troponin levels, and abnormalities detected through electrocardiograms (ECGs), echocardiograms, or cardiac magnetic resonance imaging (127, 129, 141–143). However, it’s important to note that clinical presentations can vary among cases (127, 129, 141–143). Similar to viral myocarditis, ICI-related myocarditis has been characterized by the infiltration of T cells into the myocardium (122). On a histological level, the infiltrating cells observed in cases of ICI-associated myocarditis are primarily composed of T cells and macrophages (122, 127, 143–145). Among these infiltrating T cells, CD8+ T cells tend to predominate over CD4+ T cells in instances of ICI-associated myocarditis (122, 127, 143–145). Researchers have postulated that T cells play a crucial role in the development of myocarditis associated with ICIs, given the predominant expression of PD-1 and CTLA-4 in T cells (138).

**Figure 1:**
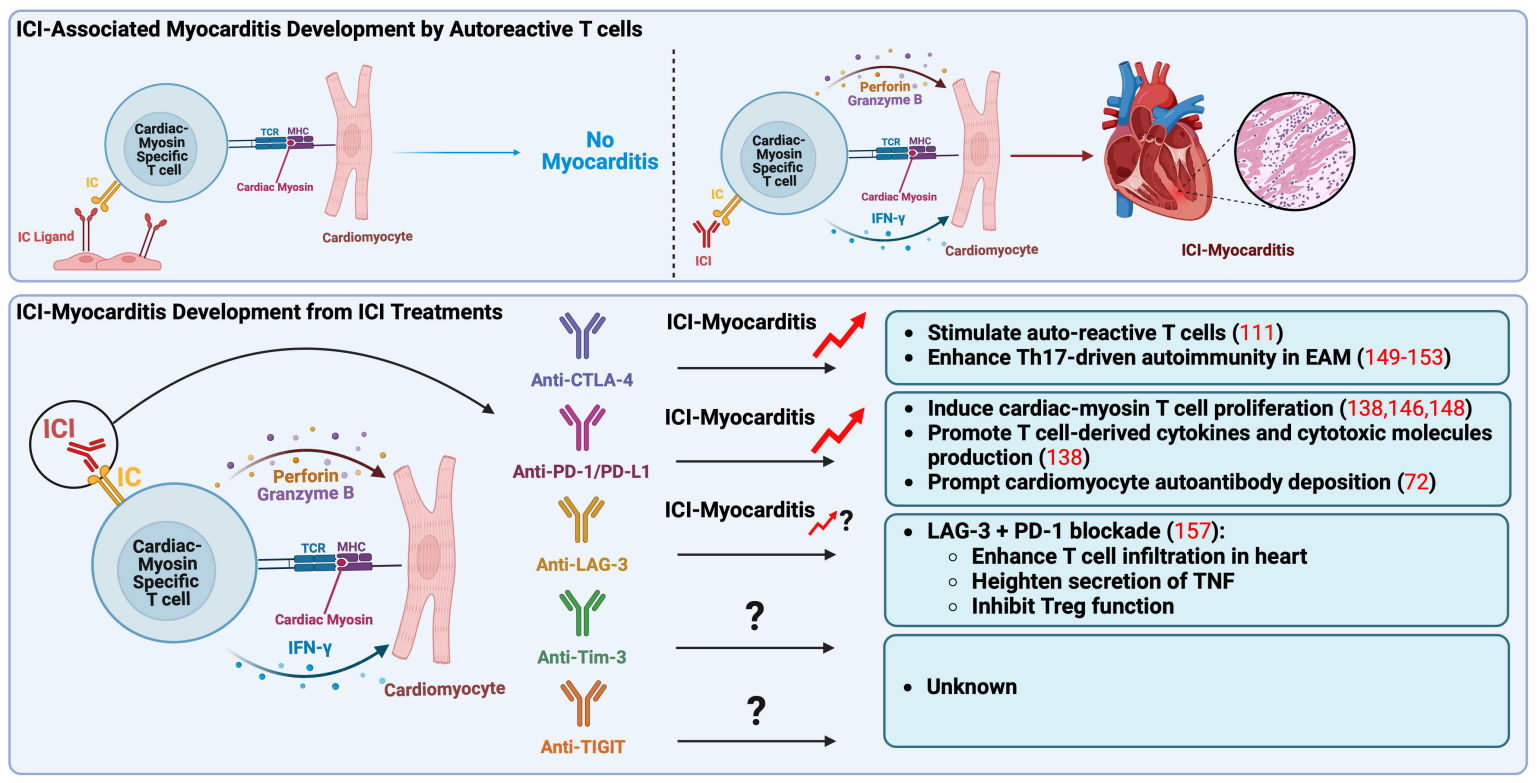
Pathogenic mechanisms of immune checkpoint inhibitors associated myocarditis. Autoreactive T cells, which recognize cardiac myosin heavy chain, main autoantigen in the heart, play a crucial role in the onset of ICI-myocarditis. These autoreactive T cells may be present in the heart in a naïve state due to impaired thymic selection, expressing elevated levels of IC as a peripheral tolerance mechanism to prevent their activation. In a mouse model, ICI treatment seems to directly activate these autoreactive T cells by obstructing the inhibitory IC pathway, specifically targeting the heart. Recent reports have indicated that anti-CTLA-4 and anti-PD-1/PD-L1 treatments result in the clonal expansion of T cells specific to cardiac myosin, observed in peripheral blood mononuclear cells of patients with ICI-associated myocarditis, indicating their pathogenic role in clinical scenarios. Anti-CTLA-4 is associated with the promotion of Th17-mediated autoimmunity in EAM, while anti-PD-1/PD-L1 is linked to excessive production of T cell derived cytokines and cytotoxic molecules like IFN-γ, perforin, and granzyme B, ultimately contributing to myocarditis development. Notably, anti-LAG-3 has only been associated with ICI-myocarditis when combined with anti-PD-1. CTLA-4, cytotoxic T-lymphocyte antigen-4; EAM, experimental autoimmune myocarditis; IC, immune checkpoint; ICI, immune checkpoint inhibitor; IFN-γ, interferon-γ; LAG-3, lymphocyte activation gene-3; MHC, major histocompatibility complex; PD-1, programmed death-1; PD-L1, programmed death ligand-1, TCR, T-cell receptor; Th17, IL-17-producing T cell (type 17 helper T cell); TIGIT, T cell immunoreceptor with immunoglobulin and ITIM domain; Tim-3, T cell immunoglobulin and mucin domain-containing protein 3; TNF, tumor necrosis factor; Treg, regulatory T lymphocytes.

In the first case report of ICI-associated myocarditis, the activation of shared T cell clones targeting both myocardium and tumor has been suggested as the mechanism of its onset and progress (122). Recently, our preclinical study revealed that autoreactive T cells specific for cardiac myosin heavy chain, a well-known heart autoantigen, lead to the development of ICI-myocarditis in mice (138). This finding is supported by an independent study reported by other researchers (146). In their study, the experimental and clinical findings involving T cells responsive to cardiac myosin heavy chain in patients imply a clear immunological relevance of cardiac myosin heavy chain expression levels in the human ventricle concerning myocarditis associated with ICIs (146). Notably, in those two studies, the development of ICI-associated myocarditis in mice did not require tumor cells, indicating that the activation of autoreactive T cells targeting the heart by an ICI treatment is sufficient to cause ICI-associated myocarditis. Furthermore, the findings from independent studies, which highlight the involvement of cardiac myosin heavy chain-reactive T cells in the development of ICI myocarditis, emphasize the critical function of immune checkpoint molecules, such as PD-1/PD-L1 and CTLA-4, in maintaining cardiac autoimmunity under normal conditions (**Figure 1**) (147).

###### 3.1.2.1.1 PD-1/PD-L1 pathway in immune checkpoint inhibitor associated myocarditis

It is reported that autoreactive T cells specific for cardiac myosin can be present in the mouse heart during a naïve state due to the impairment of thymic selection and that they express a high level of PD-1 to avoid autoimmune reactions (138, 148). In our mouse model, anti-PD-1 mAb treatment seems to directly activate autoreactive T cells targeting the heart by blocking the inhibitory PD-1/PD-L1 pathway, leading to the excessive production of effector cytokines and cytotoxic molecules such as IFN-γ, perforin, and granzyme B followed by myocardial damage and myocarditis development (138). The clonal expansion of T cells specific for cardiac myosin was observed also in the peripheral blood mononuclear cells of the patients with ICI-associated myocarditis, suggesting their pathogenic role in clinical settings (146).

###### 3.1.2.1.2 CTLA-4 pathway in immune checkpoint inhibitor associated myocarditis

Unlike the anti-PD-1 mAb treatment, it is unclear whether the anti-CTLA-4 mAb regimen mediates ICI-associated myocarditis development through the activation of autoreactive T cells targeting cardiac myosin heavy chain. However, it is reported that CTLA-4-deficient mice spontaneously develop lethal myocarditis at an early age, indicating the activation of autoreactive pathogenic T cells targeting the heart by the blockade of CTLA-4 (111). Additionally, in our mouse model for ICI-associated myocarditis, cardiac myosin-specific T cells exhibited an elevated level of CTLA-4 expression compared to bystander T cells (138). Thus, CTLA-4 inhibition using mAbs may cause fulminant myocarditis by activating autoreactive T cells targeting cardiac myosin in clinical settings as well as PD-1 inhibition.

IL-17-producing T cells, also known as Th17 cells, play a pivotal role in the pathophysiology of different types of myocarditis in both clinical and preclinical settings, as evidenced by various studies (149–152). These cells are initiators of inflammation and contribute to the progression from acute myocarditis to dilated cardiomyopathy (149–152). Notably, disrupting the interaction between CTLA-4 and B7 molecules has been shown to enhance the differentiation of Th17 cells, both in laboratory settings and in live animals, subsequently promoting Th17-mediated autoimmunity in murine models (153). Furthermore, multiple studies have indicated CTLA-4’s role in maintaining peripheral heart tolerance (42, 111, 151–155). Collectively, these findings suggest that blocking CTLA-4 pathways has the potential to contribute to the development of myocarditis and worsen the severity of the disease.

###### 3.1.2.1.3 LAG-3 pathway in immune checkpoint inhibitor associated myocarditis

Since LAG-3 has recently gained FDA approval, there is a limited number of reported cases concerning ICI-related myocarditis involving this factor. In animal experiments focusing on LAG-3, it has been previously documented that the knockout of LAG-3 in mice did not result in the onset of myocarditis (156). However, when both LAG-3 and PD-1 were knocked out, a lethal form of myocarditis emerged, characterized by T-cell infiltration, heightened secretion of tumor necrosis factor (TNF), and persistent inhibitory function of Tregs (157).

##### 3.1.2.2 Treatment for immune checkpoint inhibitor associated myocarditis

The management of ICI-associated myocarditis has predominantly relied on the administration of glucocorticoids, encompassing both oral prednisone and intravenous methylprednisolone (18, 158). According to a limited dataset, approximately 86% of patients treated with glucocorticoids exhibited improved outcomes, particularly when high-dose glucocorticoids were employed (18). Therefore, it is advisable to initiate high-dose steroid therapy at the outset, followed by a gradual tapering regimen, contingent upon clinical progress and ongoing troponin level monitoring (159). If there is an insufficient clinical or biomarker response to steroids, alternative immune modulators may be contemplated (159).

Case reports and small case series have documented successful treatment of ICI-related myocarditis with various agents, including intravenous immunoglobulin (160), mycophenolate (161), infliximab (anti-TNF mAb) (162), anti–thymocyte globulin (145), plasmapheresis (162), alemtuzumab (anti-CD52 mAb) (163), and abatacept (CTLA-4 agonistic mAb) (164). In particular, the use of abatacept either by itself or in combination with Ruxolitinib (mAb targeting IFN-γ/JAK2/STAT1 signaling pathway) has led to a notable decrease in mortality among patients suffering from ICI-associated myocarditis and myositis (165). The effectiveness of these agents in ICI-related myocarditis remains uncertain and is generally reserved for patients who do not respond adequately to glucocorticoids (159).

In addition to immunosuppressive therapy, conventional systematic therapy is essential (159). For acute decompensated heart failure, it is advisable to administer intravenous diuretics, inotropes, and mechanical circulatory support, as outlined in the American College of Cardiology/American Heart Association heart failure guidelines (166).

Due to the substantial mortality risk associated with ICI-related myocarditis, the prevailing consensus suggests discontinuing ICI therapy even in cases of mild toxicity when there is suspicion of this condition (159). Furthermore, it remains uncertain whether ICI therapy can be safely resumed following the successful treatment of ICI-related myocarditis (159). A case report indicates that resuming ICI therapy with a different agent after myocarditis developed on nivolumab resulted in worsened heart failure hospitalization with pembrolizumab (167). Consequently, it is not advisable to consider reattempting ICI therapy in patients with a history of ICI-related myocarditis (159).

#### 3.1.3 Immune checkpoint inhibitor associated atherosclerosis

Atherosclerosis represents the predominant pathological process underlying cardiovascular diseases (168). This ailment pertains to the development of atherosclerotic plaques within large- and medium-sized arteries (169). These plaques include necrotic cores, calcified regions, accumulated modified lipids, inflamed smooth muscle cells, endothelial cells, leukocytes, and foam cells (169). The intricate composition of these plaques underscores the complexity of atherosclerosis, involving numerous components of the vascular, metabolic, and immune systems (168).

Emerging data propose that ICIs might expedite the progression of atherosclerosis, potentially resulting in an elevation of atherosclerosis-related cardiovascular events like acute myocardial infarction, ischemic stroke, and peripheral arterial disease (21, 170–175). Specifically, substantial foundational cellular and animal studies indicate that the immune checkpoint proteins such as CTLA-4, PD-1, PD-L1, and LAG-3 serve as crucial negative regulators of atherosclerosis (176–178). Consequently, their inhibition could potentially accelerate atherosclerosis by intensifying effector T cell responses, constraining the function of Treg cells, and infiltrating the vascular endothelium (177–181). Moreover, a growing body of clinical evidence reinforces these preclinical discoveries by demonstrating that ICI therapy contributes to the hastened advancement of atherosclerotic plaque, consequently amplifying the susceptibility to atherosclerotic cardiovascular ailments (182–184).

In a comprehensive study, Bar et al. performed a retrospective analysis to examine the occurrence of acute vascular complications in a group of 1,215 cancer patients receiving ICI therapy (185). 1% of these patients underwent myocardial infarction or ischemic stroke within 6 months of initiating ICI treatment (185). Furthermore, a recently issued systematic review of 17 studies, comprising a total of 10,106 participants, examined the frequency of arterial thrombotic events, specifically focusing on stroke and myocardial infarction, following the administration of ICI therapy (186). They found an incidence rate of arterial thrombotic events among ICI-treated patients at 1.1% (186). Particularly, this risk appeared consistent regardless of whether patients were subjected to single or combined ICI treatment strategies (186, 187).

##### 3.1.3.1 Mechanism of immune checkpoint inhibitor associated atherosclerosis

There have been no direct mechanistic studies in humans to evaluate accelerated atherosclerosis in the setting of ICI therapy (26). However, extrapolating data from mouse models for ICI-associated atherosclerosis and ICI-myocarditis provides insight into several plausible pathways for immune checkpoint blockade to cause plaque progression (26).

**Figure 2:**
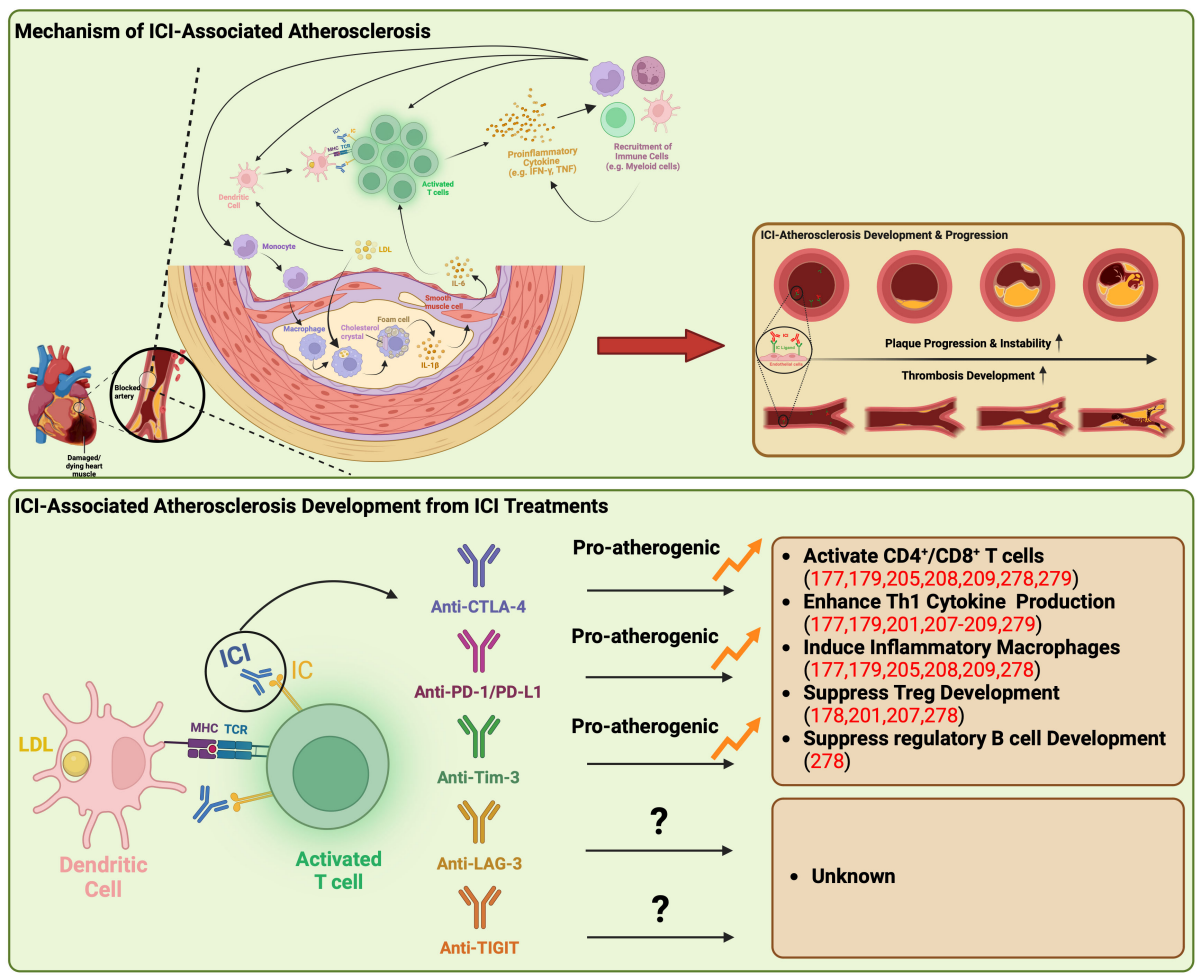
Underlying mechanisms of atherosclerosis linked to immune checkpoint inhibitors. ICI-associated atherosclerosis encompasses an intricate process of plaque formation within arterial walls, commencing with the retention of LDL-cholesterol that triggers inflammation and recruit monocytes into artery walls. These monocytes evolve into cholesterol-laden macrophages, eventually transforming into foam cells, creating a central necrotic area within the plaque. Persistent lipoprotein uptake and macrophage proliferation contribute to plaque enlargement, attracting immune cells, particularly T cells, to the plaque’s periphery. Th1 cells release IFN-γ and TNF, promoting macrophage activation and destabilizing the plaque, potentially leading to heart attacks or strokes. ICIs disrupt this process by activating T cells, producing proinflammatory cytokines like TNF and IFN-γ. These cytokines induce detrimental effects such as immune cell recruitment, smooth muscle cell proliferation, collagen deposition, and macrophage activation, triggering further proinflammatory cytokine release syndrome. This results in increased LDL phagocytosis and foam cell formation, ultimately causing structural changes within the plaque, including the formation of a necrotic core, rendering the plaque more unstable. Anti-CTLA-4, PD-1/PD-L1, and Tim-3 have been demonstrated to be pro-atherogenic, fostering the proliferation of inflammatory immune cells while suppressing the development of Treg and regulatory B cells. CTLA-4, cytotoxic T-lymphocyte antigen-4; IC, immune checkpoint; ICI, immune checkpoint inhibitor; IFN-γ, interferon-γ; IL, interleukin; LAG-3, lymphocyte activation gene-3; LDL, low-density lipoprotein; MHC, major histocompatibility complex; PD-1, programmed death-1; PD-L1, programmed death ligand-1, TCR, T-cell receptor; Th1, type 1 helper T cell; TIGIT, T cell immunoreceptor with immunoglobulin and ITIM domain; Tim-3, T cell immunoglobulin and mucin domain-containing protein 3; TNF, tumor necrosis factor; Treg, regulatory T lymphocytes.

One potential mechanism is that the revitalization of T cells by ICI may lead to excess inflammation, resulting in pro-atherogenic effects. The interaction between immune checkpoints expressed on T cells and their ligands on DCs within the microenvironment plays a crucial role in T-cell inactivation and the maintenance of an immunosuppressive microenvironment within plaques (**Figure 2**) (188). However, ICIs can disrupt this process by activating T cells, triggering the production of proinflammatory cytokines such as TNF and IFN-γ (188). These cytokines, in turn, induce various detrimental effects, including smooth muscle cell proliferation, collagen deposition, and macrophage activation (188). These changes contribute to increased phagocytosis of low-density lipoprotein (LDL) in macrophages, leading to their transformation into foam cells (173, 177). Ultimately, these structural alterations in the plaque lead to the formation of a necrotic core, rendering the plaque more unstable (188).

Another proposed mechanism for atherosclerosis associated with ICI involves the activation of T cells that recognize autoantigens specific to atherosclerosis (26). This recognition is analogous to the phenomenon observed in ICI myocarditis, where cardiac antigen-specific T cells contribute. Recent single-cell RNA analysis of human coronary atherosclerotic plaques has revealed clonal expansion of antigen-experienced T cells (189). This finding underscores a potential pathway through which self-reactive epitopes may expedite atherosclerosis by interacting with smooth muscle cells and macrophages within the plaque microenvironment (189).

###### 3.1.3.1.1 The role of CTLA-4 in atherosclerosis associated with immune checkpoint inhibitors

The interaction between CTLA-4 and CD80/CD86 binding appears to have immunoregulatory effects that suggest a protective role in atherosclerosis (190). Poels et al. conducted an assessment of the impact of antibody-mediated CTLA-4 inhibition on the progression of atherosclerosis (191). Their findings indicated a two-fold increase in the size of atherosclerotic lesions in mice treated with CTLA-4 inhibiting antibodies (191). This increase was predominantly attributed to a shift towards an activated T-cell profile, with limited alterations observed in the macrophage inflammatory profile (191). Furthermore, the inhibition of CTLA-4 was associated with the progression of plaques towards more advanced phenotypes (191). These advanced phenotypes were characterized by reduced collagen content, augmented intimal thickening, and larger necrotic core areas (191). Consistent with the observations from studies involving antibody-mediated CTLA-4 inhibition, or mice overexpressing CTLA-4 exhibited decreased intimal thickening, with a remarkable reduction of 58.5% (181, 191, 192). These mice also displayed reduced numbers of CD4 T-cells, diminished proliferation activity, and a decrease in proinflammatory cytokine production (181, 191, 192).

###### 3.1.3.1.2 The role of PD-1/PD-L1 in atherosclerosis associated with immune checkpoint inhibitors

PD-1’s ability to suppress Th1 cytokine production and facilitate Treg cell development points towards a plausible role in safeguarding against atherosclerosis (193). This protective effect of PD-1/PD-L1 activity becomes evident in knockout mice that manifest enlarged plaques characterized by heightened T-cell and macrophage populations, elevated levels of TNF, intensified T-cell activation by APCs, and amplified cytotoxicity exhibited by CD8+ T cells (177, 179). These combined factors contribute to escalated inflammation and the formation of plaques (177, 179). Additionally, the interaction of PD-1 with PD-L1 leads to the inhibition of cytokine production in differentiated Treg cells, effectively targeting Th1 cell cytokines such as IFN-γ and TNF (194). Since IFN-γ plays a pivotal role in atherogenesis by stimulating T-cell and macrophage recruitment, driving cytokine secretion, and enhancing antigen presentation by endothelial cells, the PD-1/PD-L1 binding serves to curtail both plaque size and the inflammatory response of T cells (195, 196). Similarly, in the context of humans, multiple studies have reported reduced expression of PD-1 or its ligands in individuals with coronary artery disease (CAD) and acute coronary syndrome (180, 197). This observation further substantiates the protective role of PD-1 in atherogenesis and the progression towards advanced plaque phenotypes (180, 197). Notably, within human atherosclerotic plaques, T cells exhibit elevated PD-1 expression, indicative of T-cell exhaustion in the setting of chronic inflammation (178). This complex interplay of PD-1-expressing T cells coexisting with activated counterparts within human plaques raises concerns that the inhibition of PD-1 could potentially exacerbate atherosclerosis (190).

###### 3.1.3.1.3 The role of LAG-3 in atherosclerosis associated with immune checkpoint inhibitors

Instances of atherosclerosis or CAD associated with anti-LAG-3 therapy haven’t been documented in clinical trials. Nevertheless, independent findings have shown that the presence of LAG-3 on exhausted T cells present within atherosclerotic plaques (198, 199). Additionally, an observational study conducted on the Multiethnic Study of Atherosclerosis (MESA) cohort revealed that individuals with CAD displayed elevated levels of LAG-3 (199). This study further established LAG-3 as a noteworthy predictor of CAD risk (199).

##### 3.1.3.2 Treatment for immune checkpoint inhibitor associated atherosclerosis

The treatment for ICI-associated atherosclerosis is currently in the exploratory phase (26). Statins and proprotein convertase subtilisin/kexin type 9 (PCSK9) inhibitors have proven to be safe, effective, and well-established treatments for atherosclerosis (26). However, further investigation is needed to fully characterize the risks and benefits of their application in the context of ICIs and cancer within this complex patient population (26).

Recent studies have highlighted the effectiveness of statin treatment in significantly slowing the annual progression rate of plaque volume among atherosclerosis patients (26, 183). Notably, concurrent statin use has shown promise in enhancing ICI activity, leading to increased cytotoxic CD8 T cell function and reduced pro-inflammatory cytokines (200–202). However, it is unclear if concurrent statin and ICI therapy could further elevate the low risk of statin-induced myopathy (203).

PCSK9 inhibitors, a novel class of monoclonal antibodies used in atherosclerosis treatment, operate by reducing the deviation of LDL receptors and increasing LDL-cholesterol clearance in the bloodstream (204). Alirocumab and evolocumab are two FDA-approved PCSK9 inhibitors (204). Similar to studies on statins, recent findings reveal that PCSK9 inhibitors can enhance ICI therapeutic efficacy (205–207). Studies have shown that combining PCSK9 with ICIs improves intra-tumoral cytotoxic T cell infiltration, antigen presentation, and the expression of co-inhibitory checkpoint molecules (205–207).

Despite nonspecific immunomodulatory agents, such as steroids, being associated with a lower annual rate of plaque progression, they are not recommended for preventing or suppressing ICI-induced plaque development due to their side effects (183) and the linkage of chronic glucocorticoid therapy to adverse cardiovascular outcomes (208–212).

While targeted therapies addressing the underlying immune-mediated mechanisms are still in development, the optimal management of ICI atherosclerosis should prioritize recognizing immunotherapy as a significant risk factor for atherosclerotic cardiovascular disease (26). This involves early risk stratification and optimization, emphasizing a multidisciplinary approach involving both oncologists and cardiologists to determine appropriate screening and medical management strategies (26).

## 4 Next generation immune checkpoint inhibitors: Tim-3 and TIGIT

To address the challenges posed by ICIs, researchers are exploring alternative approaches involving different immune checkpoints within the TME (6). Novel pathways and molecules are being studied with the aim of enhancing the effectiveness and broader application of immune checkpoint inhibition therapy (27). Among these options, Tim-3, also referred to as hepatitis A virus cellular receptor 2 (HAVCR2), and TIGIT, known by various names including Washington University cell adhesion molecule (WUCAM), V-set and transmembrane domain containing protein 3 (Vstm3), and V-Set and immunoglobulin domain containing protein 9 (VSIG9), have emerged as viable and promising candidates for the treatment of solid tumors (6). Ongoing clinical trials are actively investigating their therapeutic potential as immune checkpoints (6, 213). Employing antibodies to target these receptors, either independently or in conjunction with other ICIs such as anti-PD-1, has demonstrated the ability to enhance the immune response against tumors in animal models (214–227). These findings highlight the potential of Tim-3 and TIGIT as promising therapeutic targets to address the limitations observed in previous ICI treatments (6).

### 4.1 Mechanism and signaling pathways governed by Tim-3

Tim-3, formerly known as HAVCR2, belongs to the Tim gene family, which includes Tim-1 and Tim-4 (228). Tim-3 has four ligands: soluble ligands Galectin-9 (LGALS9) and high-mobility group protein B1 (HMGB1), and surface-bound ligands carcinoembryonic antigen cell adhesion molecule 1 (CEACAM-1) and phosphatidylserine (PtdSer) (229–233). Unlike other immune checkpoints, Tim-3 is exclusively upregulated by CD4+ and CD8+ cells producing IFN-γ (214, 215). Initially identified on CTLs and Th1 cells, Tim-3 inhibits type 1 immune responses, suppressing cytokine generation, including TNF and INF-γ (228, 234, 235) and is expressed in various tumor cells and immune cells including Th17, Tregs, TILs, natural killer (NK) cells, macrophages, and DCs (228, 236–243).

Understanding Tim-3 signaling pathways is incomplete, with its interactions with intracellular partners for its inhibitory function largely unexplored (234). Research suggests both inhibitory and stimulatory roles for Tim-3 (234). Tim-3 lacks conventional inhibitory signaling motifs and structural elements for recruiting inhibitory phosphatases in its cytoplasmic tail (244). It has five conserved tyrosine residues, with phosphorylation of Tyr256 and Tyr263 (Tyr265 and Tyr272 in humans) being crucial (234). In the current model, upon T-cell activation, Tim-3 migrates to lipid rafts and interacts with BAT3 and LCK (245, 246). In the absence of ligand binding, BAT3 remains bound to Tim-3’s tail, recruiting an active form of LCK, promoting T-cell proliferation and survival (246, 247). However, ligand engagement displaces BAT3, allowing recruitment of tyrosine phosphatases (CD45 and CD148), inactivating LCK, downregulating TCR signaling, suppressing T-cell proliferation and survival (247). Galectin-9 and CEACAM-1 induce phosphorylation of Tyr256 and Tyr263 by ITK, leading to BAT3 release, allowing Tim-3 to exert its inhibitory function (232, 248). BAT3-mediated regulation of Tim-3 signaling is associated with inhibitory actions towards T cells, and its potential application in other cell types like DCs is under investigation (247).

### 4.2 Mechanism and signaling pathways governed by TIGIT

TIGIT, previously known as Vstm3, VSIG9, or WUCAM, is characterized by a protein structure with an extracellular IgV domain and an intracellular domain containing both a canonical ITIM and an immunoglobulin tyrosine tail (ITT) motif (249, 250). Its expression is specifically limited to lymphocytes, prominently observed in subsets such as NK cells, effector and regulatory CD4+ T cells, follicular helper CD4+ T cells, and effector CD8+ T cells (217, 250–254). Notably, TIGIT is found in various cancers, including melanoma, NSCLC, colon cancer, HCC, gastric cancer, glioblastoma, and hematological malignancies (218–220, 255–263). Moreover, TIGIT is significantly overexpressed by Tregs in the peripheral blood mononuclear cells of both healthy individuals and cancer patients, with further upregulation within TME (255, 264).

TIGIT exhibits binding affinity for two members of the nectin family, CD155 (poliovirus receptor, Necl-5), and CD112 (poliovirus receptor related-2, Nectin-2) (249). Its affinity for CD155 (Kd=1-3 nM) surpasses its affinity for CD112 (Kd remains unmeasurable) (249). CD112 has a stronger affinity for CD112R (PVRIG) than for TIGIT, primarily suppressing T cells through binding to CD112R rather than TIGIT (265–267). Consequently, TIGIT’s modulation of T cell and NK cell functions mainly occurs through its interaction with CD155 (251).

Functionally, TIGIT has the ability to hinder CD8+ T cell proliferation and activation by directly influencing TCR expression, leading to the downregulation of the TCR-α chain and other components forming the TCR complex (268). Additionally, TIGIT can curtail TCR-induced p-ERK signaling within CD8+ T cells, thereby suppressing CD8+ T cell priming, differentiation, and cytotoxic activity (269). Similar to CTLA-4’s inhibition of CD28’s co-stimulatory interaction with shared ligands CD80 and CD86, TIGIT exerts its effects indirectly (251). It competes for ligand binding with CD226 (DNAM-1), consequently diminishing T-cell co-stimulation through CD226 (253). Furthermore, TIGIT can obstruct co-stimulatory signaling via CD226 by impeding CD226’s homo-dimerization (217). Ultimately, TIGIT indirectly suppresses T cells by modulating the functions of cells expressing its ligand CD155 (251).

### 4.3 Application of Tim-3 and TIGIT blockade in clinical settings

#### 4.3.1 Clinical effects of Tim-3 blockade in cancer treatment

Extensive evidence from studies utilizing preclinical cancer models and *in vitro* cultures using patient samples has provided substantial support for considering Tim-3 inhibition as a promising avenue for enhancing antitumor immunity (214, 216, 220, 221). The advancement of Tim-3 as an immunotherapeutic target has been strengthened by results similar to those seen with PD-1 inhibitors (221). Experimental evidence showcases that obstructing Tim-3 expression leads to enhanced T cell proliferation and increased cytokine production, thereby stimulating immune activation (215). Furthermore, it has been documented that heightened Tim-3 levels are linked to the emergence of resistance against PD-1 blockade, evident in both lung cancer patient samples and lung cancer models, as well as samples taken from patients with head and neck cancer (222, 223). This intriguing observation implies that Tim-3 might serve as a viable alternative for patients exhibiting resistance to PD-1 blocking antibodies (222). Moreover, in preclinical settings, the combination treatment targeting Tim-3 and PD-1 has exhibited a synergistic effect, reinvigorating T cell function and augmenting the overall anti-tumor immune response (187, 195). Consequently, the concurrent blockade of PD-1 and Tim-3 emerges as a promising and feasible therapeutic strategy (214, 216, 221, 222, 224, 270–274). Currently, there are seven mAbs targeting Tim-3 and one bispecific antibody targeting both PD-1 and Tim-3 (RO7121661) that are undergoing clinical trials (247).

#### 4.3.2 Clinical effects of TIGIT blockade in cancers treatment

The outcome of single TIGIT blockade has exhibited limited or moderate antitumor effectiveness in experimental tumor models and in enhancing the *in vitro* functionality of human tumor-infiltrating CD8+ T cells (217, 218, 220, 225, 275). Nonetheless, it’s noteworthy that the interaction between CD155 and TIGIT plays a role in conferring resistance to PD-1 blockade within the context of cancer (226, 276). Studies have indicated that *in vitro* PD-1 blockade leads to an increase in TIGIT expression on NY-ESO-1-reactive CD8+ T cells extracted from melanoma patients (219). Additionally, TIGIT emerges as the most prominently upregulated immune checkpoint on CD8+ TILs following anti-PD-1 treatment in a PD-1 non-responsive HCC mouse model (275). This observation suggests that inhibiting TIGIT might hold promise as a strategy to enhance the efficacy of PD-1 blockade therapy, particularly against tumors that have developed resistance to PD-1 inhibitors (275). Furthermore, preclinical studies have both demonstrated and mechanistically elucidated the synergy between TIGIT blockade and PD-1 blockade in augmenting the antitumor CD8+ T cell response (226, 227, 275). These findings underscore the potential of combining PD-1 and TIGIT blockade as a promising approach to overcome resistance observed with single PD-1/PD-L1 blockade (226, 227, 275). In the context of mice carrying HCC and displaying resistance to PD-1 blockade, the dual inhibition of TIGIT and PD-1 has been shown to expand the population of effector memory CD8+ T cells and elevate the ratio of CTLs to Tregs within tumors (275). This, in turn, resulted in the suppression of tumor growth and extended survival (275, 277). Moreover, in an MC38 colon tumor mouse model, animals treated with dual anti-TIGIT/PD-1 antibodies exhibited heightened secretion of IFN-γ by CD4+ TILs in comparison to those treated solely with anti-PD-1 therapy (277). Presently, an array of nine human anti-TIGIT mAbs are undergoing evaluation in a total of 43 Phase 1/2/3 clinical trials (251). These trials include monotherapy usage, but more frequently involve combinations with anti-PD-1/PD-L1 antibodies or chemotherapies (251).

### 4.4 Cardiotoxic immune-related adverse events from Tim-3/TIGIT blockade

#### 4.4.1 Tim-3 pathway in cardiovascular immune-related adverse events

As investigations into the safety of Tim-3 blockade continue, there is a limited number of reported cases regarding cardiac toxic adverse events associated with anti-Tim-3 treatment. Nevertheless, a recent report has proposed that Tim-3 may function as a negative regulator or atherosclerosis (278). This particular study has demonstrated that anti-Tim-3 treatment enhances the development of atherosclerotic lesions, leading to an increased presence of monocytes, macrophages, and CD4+ T cells, while simultaneously reducing Tregs and regulatory B cells (278). Furthermore, an independent study has observed that co-blockade of PD-1 and Tim-3 results in a decrease in the production of anti-atherogenic cytokines by PD1+ Tim-3+ CD8+ T cells and an increase in the production of TNF and IFN-γ, both of which contribute to the development and progression of atherosclerosis (279).

#### 4.4.2 TIGIT pathway in cardiovascular immune-related adverse events

While clinical trials have not reported any occurrences of cardiac toxic irAEs linked to anti-TIGIT therapy so far, ongoing investigations are actively exploring the potential emergence of cardiac toxic irAEs resulting from TIGIT blockade treatment. This exploration is particularly focused on conditions characterized by elevated TIGIT expression, especially in cardiovascular diseases. The heightened expression of TIGIT ligands raises the question of whether it signifies a potential risk of cardiotoxic irAEs or suggests a protective function of TIGIT against cardiovascular diseases. This underscores the need for further research to elucidate the potential association between anti-TIGIT treatment and cardiac toxic irAEs.

## 5 Conclusions and future directions

In cancer treatment, inhibitory targeting of CTLA-4 and PD-1/PD-L1 has evolved into a fundamental approach (27). Despite the proven viability and effectiveness of these ICIs, significant obstacles persist within the field of cancer immunotherapy (6). These hurdles include a restricted response rate and the occurrence of irAEs (6). Notably, among these challenges, the impact on the heart is particularly concerning, with instances of severe cardiac toxicity and a growing number of cases linking CAD to ICIs (91, 218). These challenges underscore the pressing requirement for innovative tactics to mitigate the adverse effects associated with therapy. In response to these challenges, there has been a gradual diversification in the array of co-inhibitory receptor pathways, extending beyond the established CTLA-4 and PD-1 pathways (280). Particularly noteworthy is the incorporation of LAG-3, Tim-3, and, more recently, TIGIT, which has significantly enriched the existing repertoire of approaches aimed at addressing these obstacles (280).

While the occurrence of cardiovascular adverse events related to ICIs is relatively infrequent, their associated mortality rate is notably high (281). Recently, several cardiovascular irAEs have come to light, encompassing conditions such as myocarditis, atherosclerosis, pericarditis, arrhythmias, and cardiomyopathy (123). Among these, myocarditis has exhibited a significant increase in its association with ICIs in recent years and is particularly concerning due to its significantly elevated mortality rate ranging from 25 to 50% (13, 15, 16, 18, 122, 131, 132, 140). Our recent preclinical study has illuminated the crucial role of ICIs like PD-1/PD-L1 and CTLA-4 in maintaining cardiac autoimmunity under normal conditions, emphasizing their importance in this context (138). Furthermore, emerging data suggest that ICIs may expedite the progression of atherosclerosis, potentially leading to an increased risk of atherosclerosis-related cardiovascular events such as acute myocardial infarction, ischemic stroke, and peripheral arterial disease (173–176). Notably, the blockade of the CTLA-4 and PD-1/PD-L1 pathways is significantly associated with the occurrence and progression of both myocarditis and atherosclerosis (42, 138, 146, 148, 151–155, 178–181, 191–193, 195–198, 282). While combining ICIs had significantly improved therapeutic effectiveness, it also carries a considerably higher risk of cardiac irAEs, resulting in increased incidence and mortality rates compared to single-agent therapy (5, 15, 117, 121, 122).

While ongoing clinical trials are actively evaluating the therapeutic efficacy and safety of next-generation ICIs like LAG-3, Tim-3, and TIGIT, Anderson and colleagues have put forth a hierarchical model illustrating the role of co-inhibitory receptors (280). This model proposes that LAG-3, Tim-3, or TIGIT could potentially serve as safer alternatives to existing ICIs based on their roles in regulating self-tolerance and autoimmune toxicity, with CTLA-4 and PD-1 being the primary regulators of self-tolerance and LAG-3, Tim-3, and TIGIT forming a second tier of co-inhibitory molecules with distinct roles in immune response regulation. Furthermore, the latest generation of ICIs offers the potential for targeted regulation of immune responses within specific tissue environments, leveraging the expression of their corresponding ligands to uphold tissue tolerance and prevent immune-related damage (280). This concept underscores the proposed tissue-specific immunoregulatory roles of LAG-3, Tim-3, and TIGIT.

However, it is important to note that increased levels of these ligands must not be exclusively construed as a conclusive sign that blocking these ICIs or their ligands would inherently lead to significant therapeutic advantages. Heightened ligand expression might stem from an inflammatory response triggered by the disease, or it could play a role in a defensive mechanism against the disease. Consequently, pursuing this strategy might potentially trigger serious irAEs that carry the potential for life-threatening outcomes. Moreover, as previously mentioned, the majority of clinical trials have underscored that the use of standalone therapy involving the new generation ICI does not exhibit notable therapeutic effectiveness when contrasted with the outcomes of anti-CTLA-4 or PD-1 treatments—except when combined with anti-CTLA-4 or PD-1 agents (101, 214, 216, 221, 222, 224, 226, 227, 270, 271, 273–275). As a result, further investigation is necessary to unveil the precise therapeutic efficacy and safety profile of these next-generation ICIs. This pursuit could potentially establish them as secure alternatives for ICI-based cancer treatments.

## Author contributions

WJ: Conceptualization, Formal analysis, Investigation, Visualization, Writing – original draft, Writing – review & editing. TW: Conceptualization, Investigation, Writing – original draft, Writing – review & editing. AD: Formal analysis, Investigation, Resources, Visualization, Writing – review & editing. DČ: Conceptualization, Project administration, Resources, Supervision, Writing – review & editing.
